# MERS-CoV and SARS-CoV-2 infection in diverse human lung organoid-derived cultures

**DOI:** 10.1128/jvi.01098-25

**Published:** 2025-09-18

**Authors:** Kim Lam Chiok, Kristof Jenik, Mark Fenton, Darryl Falzarano, Neeraj Dhar, Arinjay Banerjee

**Affiliations:** 1 Vaccine and Infectious Disease Organization (VIDO), University of Saskatchewan 7235, Saskatoon, Saskatchewan, Canada; 2 Division of Respirology, Critical Care and Sleep Medicine, Respiratory Research Centre, University of Saskatchewan 7235, Saskatoon, Saskatchewan, Canada; 3 Department of Veterinary Microbiology, Western College of Veterinary Medicine, University of Saskatchewan 7235, Saskatoon, Saskatchewan, Canada; 4 Department of Biochemistry, Microbiology, and Immunology, University of Saskatchewan 7235, Saskatoon, Saskatchewan, Canada; 5 School of Public Health, University of Saskatchewan 7235, Saskatoon, Saskatchewan, Canada; 6 Department of Biology, University of Waterloo 98689, Waterloo, Ontario, Canada; 7 Department of Biochemistry and Molecular Biology, University of British Columbia 8166, Vancouver, British Columbia, Canada; 8 Department of Laboratory Medicine and Pathobiology, Temerty Faculty of Medicine, University of Toronto 12366, Toronto, Ontario, Canada; Loyola University Chicago - Health Sciences Campus, Maywood, Illinois, USA

**Keywords:** coronavirus, SARS-CoV-2, MERS-CoV, tissue culture models, patient-derived models, respiratory disease, virus-host interactions, high containment, airway organoid, air-liquid interface cultures

## Abstract

Cell cultures are widely used to study infectious respiratory diseases and to test therapeutics; however, they do not faithfully recapitulate the architecture and complexity of the human respiratory tract. Lung organoids have emerged as an alternative model that partially overcomes this key disadvantage. Lung organoids can be cultured in various formats that offer potential for studying highly pathogenic viruses. However, the effects of these different formats on virus infection remain unexplored, leaving their relative value unclear. In this study, we generated primary lung organoids from human donor cells and used them to derive monolayers and air-liquid interface (ALI) cultures with the goal of comparing the replication kinetics of two circulating highly pathogenic coronaviruses, severe acute respiratory syndrome coronavirus 2 (SARS-CoV-2) and Middle East respiratory syndrome coronavirus (MERS-CoV). Infection studies revealed that organoid-derived monolayers displayed limited infection, and the innate immune response was impaired against bacterial lipopolysaccharide (LPS) but not against virus-like double-stranded RNA (dsRNA) or poly(I:C). Meanwhile, organoids and organoid-derived ALI cultures retained viral permissivity, with ALI cultures displaying diverse antiviral immune responses against both coronaviruses. SARS-CoV-2 and MERS-CoV demonstrated differential replication kinetics in organoid and organoid-derived ALI cultures. Therefore, primary organoid-derived cells in two-dimensional monolayer or three-dimensional ALI formats influence virus infection and host antiviral responses. Our study informs the selection of culture conditions for organoid-based respiratory disease research and therapeutic testing.

**IMPORTANCE:**

The COVID-19 pandemic heralded the upsurge in human-derived lung organoid-based studies due to their cellular heterogeneity that partly emulates the cellular complexity of the respiratory tract. A major disadvantage of organoid models resides in their apical-in conformation that “hides” cells and proteins that are typically exposed to the air-liquid interface (ALI) in the airways and are targets of viruses. Here, we generated monolayers and ALI cultures to facilitate cell exposure to highly relevant pathogens and compared them to parental organoids. Organoids at the ALI captured infection and immune responses better than organoids and organoid-derived monolayer cultures. Organoids at the ALI are a viable approach to improve identification and characterization of virus infection, host responses, and therapeutic testing.

## INTRODUCTION

Traditionally, researchers have relied on two-dimensional (2D) cell culture models such as immortalized cell lines to uncover molecular mechanisms of disease, understand the host immune response against infectious viral agents, identify therapeutic targets, and assess drug efficacy (1). Even for studying respiratory viruses, cells are typically cultured as 2D monolayers, submerged in nutrient-rich media that supports their growth and replication. While these cell lines are important tools for studying disease mechanisms, they often fail to replicate the cellular heterogeneity, three-dimensional architecture, and physiological responses of respiratory tissue, which is naturally exposed to environmental air in the airways. Animal models are useful to validate *in vitro* observations; however, these models can be ethically challenging and yield inconclusive outcomes due to anatomical and cellular differences when compared to humans. This is a major bottleneck when studying newly emerging viruses where time is critical and the absence of validated animal models impedes the evaluation of therapeutics⁠ (2). As emerging pathogens are a continuous threat to global health, human-like *in vitro* models are key to identify, develop, and test prophylactic and therapeutic measures.

Organoids derived from human cells have emerged as an alternate model that partly recapitulates the structural and compositional complexity of the native organ. Organoids are composed of self-renewing stem cells that differentiate into various cell types present in the tissue of origin and self-assemble into three-dimensional (3D) microtissues (3). Adult stem cells (ASC) from donor tissue are viable sources for organoid development without the lengthy differentiation process used for induced pluripotent stem cells (iPSCs) or the oncogenic mutations of cancer-derived organoids (4). Donor-derived organoids preserve the individual-level diversity that influences immune response, susceptibility to pathogens, metabolism, and tolerance to pharmaceuticals. These advantages have poised organoids as promising approaches for use in preclinical therapeutic screening and have received authorization from the US Food and Drug Administration (FDA) for this purpose (5). Human respiratory organoids are being actively used to study respiratory pathogens like influenza A virus (IAV) ⁠ (6), respiratory syncytial virus (RSV) (7), human adenovirus type 3 (HAdV-3) and type 55 (HAdV-55) (8), and the recently emerged coronaviruses, severe acute respiratory syndrome coronavirus 2 (SARS-CoV-2) and Middle East respiratory syndrome coronavirus (MERS-CoV) (9, 10). Despite this progress, studies comparing 2D and 3D cell culture models and their impact on SARS-CoV-2 and MERS-CoV replication are limited, particularly in the case of the highly pathogenic MERS-CoV.

SARS-CoV-2 emerged in late 2019 to cause the COVID-19 (coronavirus disease 2019) pandemic. As of December 2024, the World Health Organization (WHO) has reported over 7 million deaths worldwide since the COVID-19 pandemic started, which is likely an underestimate of the true impact of this pandemic ⁠ (11). COVID-19 mortality rates are estimated to be between 0.1% and 5% (12). The WHO data show that the virus continues to disseminate, propelled by the emergence of viral variants that can at least partially evade protection from existing vaccines ⁠ (13). MERS-CoV emerged in 2012 and continues to cause outbreaks of severe viral pneumonia with an approximate case fatality rate of 35% (14). MERS-CoV remains a pathogen of concern and a pandemic threat. Indeed, research into SARS-CoV-2 and MERS-CoV can provide insights into coronavirus biology which will inform the development of prophylactic and therapeutic interventions. Human-derived experimental models are, thus, essential to understand and mitigate risks posed by emerging coronaviruses and other respiratory pathogens.

In this study, we established human donor-derived lung organoids from which we derived traditional cell monolayers and air-liquid interface (ALI) cultures and compared infectivity using currently circulating highly pathogenic coronaviruses, SARS-CoV-2 and MERS-CoV. We used protocols, supplies, and equipment that are widely available in virology laboratories to promote the adoption of 3D systems for virological studies. We generated different 3D culture systems from the same genetic background to facilitate comparison across systems and inform the criteria for selection of models for virology research. We also aimed to expand work on MERS-CoV in 3D models due to its relevance as a virus with pandemic potential and the scarcity of studies that have used 3D cultures to study this pathogen. Lung organoids and derived monolayers and ALI cultures showed distinct differences in virus infection and transcriptional regulation of antiviral genes upon immune stimulation and virus infection. While monolayers transitioned into a virus-resistant phenotype, ALI cultures sustained viral infection and antiviral response against virus infection. Despite their common origin, differences between two- (2D) and three-dimensional (3D) cultures emphasize the need for careful selection of cell culture models for respiratory infectious disease studies and therapeutic testing.

## RESULTS

### Cell culture platforms influence organoid growth properties

**Fig 1:**
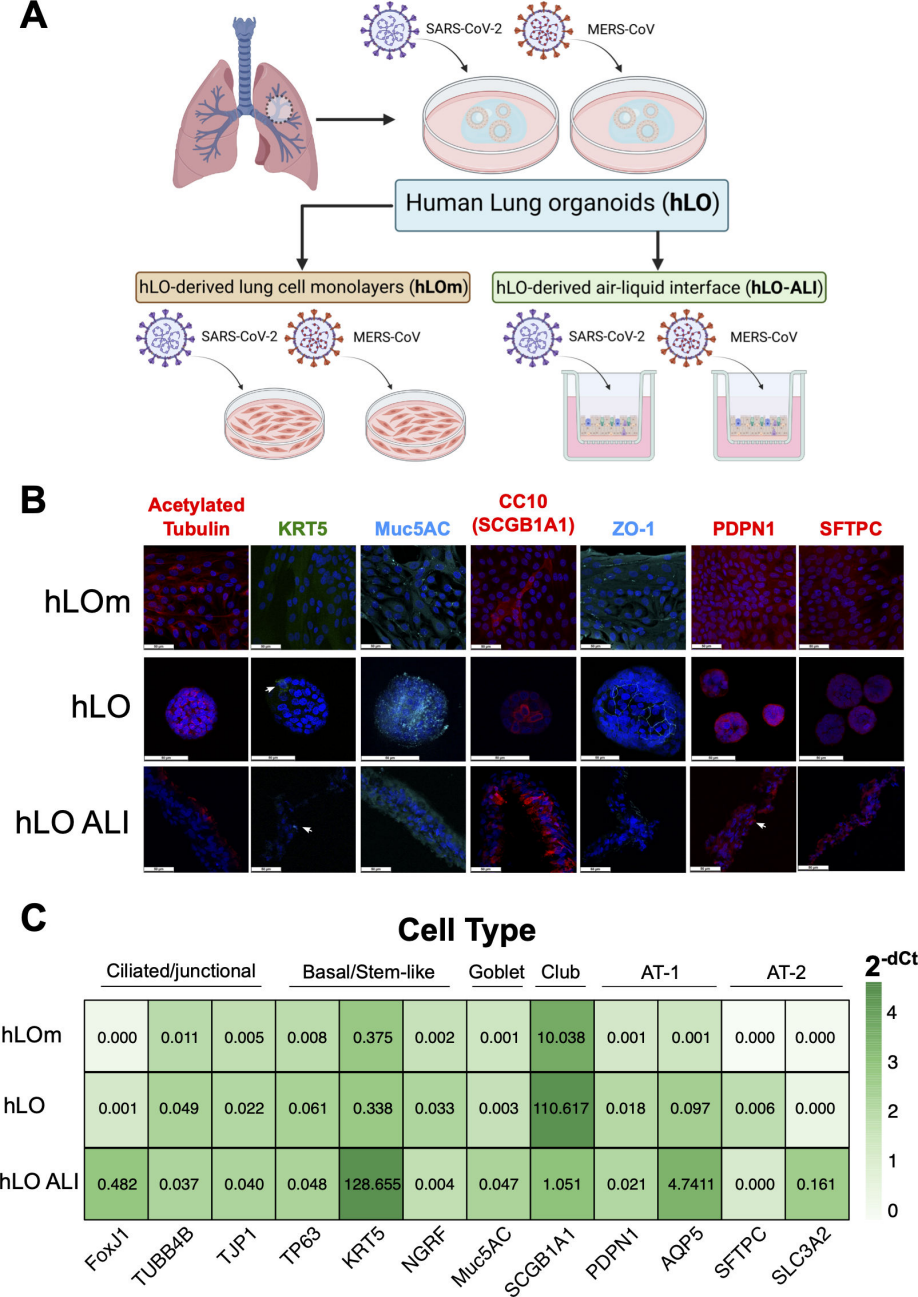
Generation of donor-derived lung organoids (hLO), organoid-derived monolayers (hLOm), and ALI (hLO ALI) cultures. (**A**) Schematic of hLO generation and derivation of hLOm and hLO-ALI cultures, followed by infection with SARS-CoV-2 or MERS-CoV. Schematic created using BioRender. (**B**) Identification of lung cell markers. hLOm, hLO, and hLO ALI cells were fixed in 4% paraformaldehyde, permeabilized and stained with antibodies against acetylated tubulin (cilia), KRT5 (Basal Stem Cells, white arrows), Muc5AC (mucus and mucus producing cells, Goblet Cells), CC10 (Club Cells), ZO-1 (zona occludens-1, tight junctions), PDPN1 (podoplanin-1, AT-1 cells, white arrows), SFTPC (surfactant protein C, AT-2 cells), and DAPI (nuclei, blue). Cells were imaged on a confocal microscope using a 63× objective. Scale bars correspond to 50 µm. (**C**) Gene expression of lung markers *FoxJ1*, *TUBB4B*, *TJP1*, *TP63*, *KRT5*, *NGRF*, *Muc5AC*, *SCGB1A1*, *PDPN1*, *AQP5* (aquaporin 5, AT-1 cells), *SFTPC* and *SLC34A2* (AT-2 cells) in hLO, hLOm, and hLO ALI was determined by RT-qPCR assays. Samples were assayed in technical duplicates, dCt was normalized to GAPDH, and expression was calculated by 2^−dCt^. The mean of three independent samples is presented, and error bars are standard error of the mean (SEM).

We obtained lung tissue sample from a donor patient and used previously established protocols ⁠ (15) to generate human lung organoids (hLO). These hLO were then cultured and maintained under three different formats—traditional two-dimensional adherent monolayers (hLOm); as three-dimensional air-liquid interface cultures (hLO ALI) or passaged as three-dimensional lung organoids (hLO) (Fig. 1A).

To characterize the distribution of different cell types in the hLOm and hLOs, we carried out immunofluorescence staining using antibodies directed to cell type-specific markers (Fig. 1B). Acetylated tubulin localized along cytoplasmic microtubules in hLOm, within the hLOs indicating an apical-in orientation, and consistent with cilia formation in the apical-out orientation in hLO ALI cultures (Fig. 1B). We identified the mucus component, Muc5AC produced by Goblet cells (16), and the tight junction protein, zona occludens-1 (ZO-1) in hLO and hLO ALI cultures identified as individual cells stained with corresponding markers. Club cells (CC10) typical of bronchiolar epithelium (17) were more abundant in hLOs and hLO ALI relative to hLO monolayers. In hLO ALI, CC10 cells appeared containing secretory granules that suggested functional production and secretion of surfactant-like glycoproteins (Fig. 1B). We did not identify specific individual cells positive for Surfactant Protein C (SFTPC) in any of the cultures, suggesting limited presence of alveolar type 2 (AT-2) cells (Fig. 1B). Similarly, we observed weak staining of KRT5+ cells (basal stem cells) and the alveolar type 1 (AT-1) protein, podoplanin-1 (PDPN1). The absence of several markers in hLOm suggested that this format did not foster various cell types and structures typical of the lungs (Fig. 1B).

We also employed RT-qPCR assays to further investigate the composition of hLOs and the derived culture models (Fig. 1C). We found that markers for ciliated, upper barrier cells (*FoxJ1, TUBB4B, TJP1*) were expressed in 3D format hLO and hLO ALI. Transcript levels of *KRT5* (marker for basal cells) were higher in hLO ALI, consistent with ALI promotion of basal stem cell growth reported previously (18). Expression of stem cell-like markers like *TP63* (tumor protein 63) and *NGRF* (Neurogranin) mirrored each other and were mostly expressed in hLO, suggesting differentiation processes in hLO ALI that correlate with loss of stem cell like properties. *Muc5AC*, a marker of Goblet cells was predominant in hLO ALI, suggesting that this system can produce mucus. *CC10* (or *SCGB1A1*) transcripts were expressed in all three systems, indicating the potential secretion of glycoproteins by club cells typical of the bronchiolar epithelium (Fig. 1C).

We determined transcript levels of *AQP5* (*aquaporin 5*) and *PDPN1* as markers for AT-1 cells. The early alveolar structural marker, *PDPN1* remained low in both 3D systems. In contrast, *AQP5* was predominantly expressed in hLO ALI cultures, suggesting potential metabolic activity associated with water export and hydration of the epithelium. Transcripts for the AT-2 cellular marker, *SFTPC* was almost undetectable in 3D models, while transcripts for *SLC34A2* (encoding a phosphate transporter involved in surfactant synthesis) was predominant in hLO ALI (Fig. 1C). Therefore, our data demonstrate that cellular composition was different between hLO, hLOm, and hLO ALI cultures derived from the same parental organoids. Our results suggest that 3D formats like hLO and hLO ALI consist primarily of lung bronchial airway cells that are absent in cell culture monolayers.

### Culture format influences innate immune response against virus- and bacteria-like stimuli

**Fig 2:**
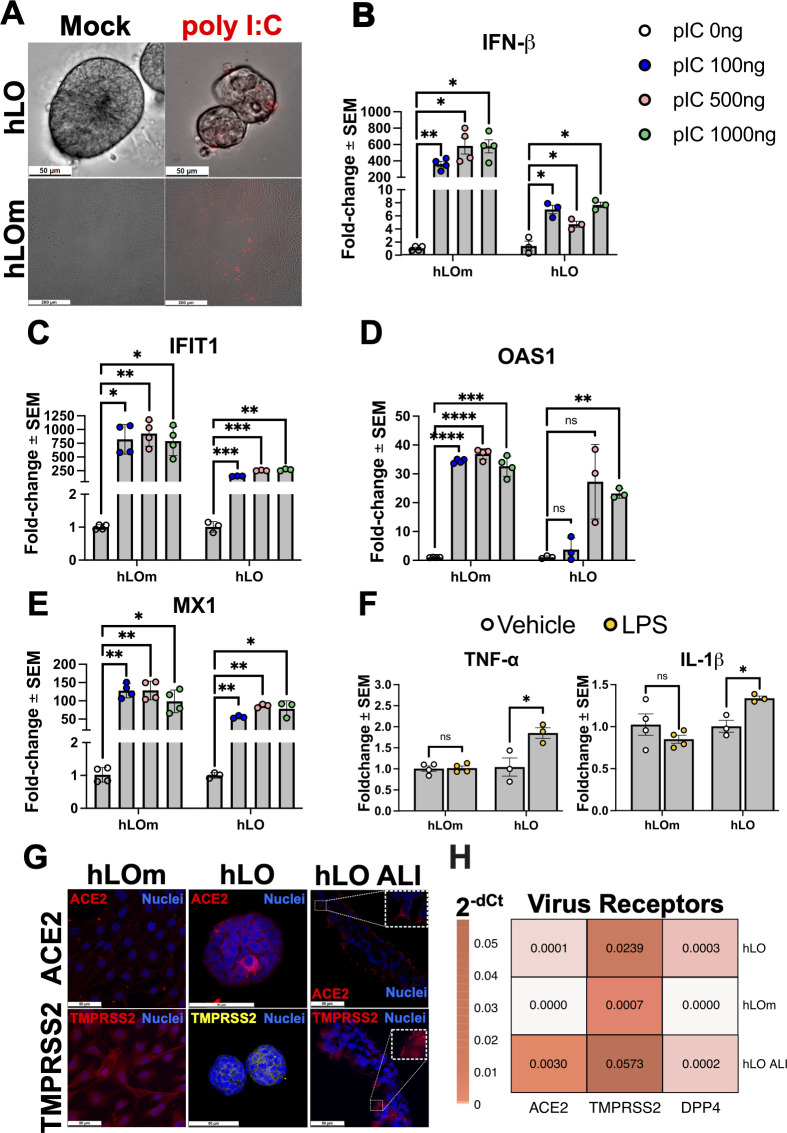
Evaluation of immunocompetence against virus- and bacteria-like stimuli. (**A**) hLO and hLOm were mock-transfected or transfected with 2 µg of rhodamine labeled polyI:C (red). Live cells were imaged at 48 h post transfection. Scale bars correspond to 50 µm (hLO, top panels) and 200 µm (hLOm, bottom panels). (**B**) hLO and hLOm were transfected with increasing doses of polyI:C (pIC) for 48 h. Total RNA was collected for RT-qPCR assays to determine gene expression of *IFNβ* (**B**)*, IFIT1* (**C**), *OAS1* (**D**), and *MX1* (**E**). (**F**) hLO and hLOm were treated with either vehicle (LPS-free water) or LPS (100 ng/mL) for 6 h. RNA was collected for RT-qPCR determination of *TNF-α* and *IL-1β* transcript levels. (**G**) hLOm, hLO, and hLO ALI were fixed in paraformaldehyde 4%, permeabilized and stained with antibodies against ACE2 and TMPRSS2, and counterstained with DAPI (nuclei, blue). Cells were imaged using a confocal microscope at immersion (63×). Individual positive cells are presented as insets highlighted with white boxes. Scale bars correspond to 50 µm. (**H**) Gene expression levels of *ACE2*, *TMPRSS2* and *DPP4* in hLO, hLOm, and hLO ALI were determined by RT-qPCR assays. Samples were assayed in technical duplicates, dCt was normalized to *GAPDH,* and expression was calculated by 2^−dCt^. The mean of three independent samples is presented. For RT-qPCR assays in B to F, samples were assayed in technical duplicates, dCt was normalized to GAPDH, and expression was calculated as fold-change relative to vehicle treated controls by 2^−ddCt^ method. The mean of three independent samples is presented, and error bars are standard error of the mean (SEM). Empty circles = vehicle or 0 ng; Blue circles = polyI:C 100 ng; Pink circles = polyI:C 500 ng; Green circles = polyI:C 1 µg; Yellow circles = LPS 100 ng/mL. Statistical analysis was performed by One-way ANOVA (**B–E**) or Student’s *t* test (**F**). *, *P* < 0.05; **, *P* < 0.01; ***, *P* < 0.001; ****, *P* < 0.0001.

Next, we examined the cellular response against bacteria-like and virus-like stimuli in hLOm and hLO as prototypic 2D and 3D culture systems. hLOm and hLO were transfected with multiple doses of the dsRNA analog polyinosinic-polycytidylic acid [poly(I:C)] to mimic viral infection. Poly(I:C) was delivered successfully in both hLOm and hLO as observed by live cell imaging (Fig. 2A). hLOm and hLO responded to this stimulus by upregulating the transcripts for antiviral genes like interferon-beta (*IFNβ*) (Fig. 2B), interferon Induced Protein with tetratricopeptide 1 (*IFIT1*) (Fig. 2C), 2′−5′-oligoadenylate synthetase 1 (*OAS1*) (Fig. 2D), and MX Dynamin Like GTPase 1 (*MX1*) (Fig. 2E). While most responses were comparable between hLO and hLOm, upregulation of *IFNβ* was nearly 100-fold higher in hLOm (Fig. 2B). We (19) and others (20) previously identified *IFN*β and this set of interferon-stimulated genes (ISGs) as relevant for antiviral responses against coronaviruses. Therefore, both hLO and hLOm upregulate antiviral genes upon encountering intracellular virus-like stimuli.

Lipopolysaccharide (LPS) is a major component of the outer membrane of gram-negative bacteria that induces production of tumor necrosis factor alpha (*TNF-α*) and interleukin 1 beta (*IL-1β*) to mediate inflammation and acute lung injury in cellular and animal models (21). hLOs exposed to LPS from pathogenic *E. coli* O111:B4 responded by upregulating the proinflammatory genes *TNF-α* and *IL-1β* (Fig. 2F). In contrast, hLOm did not upregulate these proinflammatory cytokines and remained unresponsive to LPS stimuli. These results indicate that donor-derived hLO and corresponding hLOm are immunocompetent and responsive to virus-like stimuli, but only hLOs respond to LPS. Thus, transition from 3D to 2D culture formats may influence the breadth of the innate immune response, with hLOm missing relevant pulmonary responses such as those directed against bacterial LPS.

Next, we aimed to map the distribution of coronavirus receptors in hLOm, hLO, and hLO ALI. Immunofluorescence staining revealed that distinct cells within parental hLOs expressed angiotensin converting enzyme 2 (ACE2) and TMPRSS2, the cellular receptor for SARS-CoV-2, and its entry cellular co-factor, respectively (22) (Fig. 2G). We did not detect ACE2 in monolayers, whereas TMPRSS2 appeared less intense and diffuse in cytoplasms of cells in monolayers (Fig. 2G). Individual cells in hLO ALI also expressed ACE2 and TMPRSS2, the latter of which localized to the cell membrane and cilia (Fig. 2G), similar to what has been reported previously for primary cell nasal epithelium ALI cultures (23). Despite our best efforts, we were unable to detect the DPP4 protein, the receptor for MERS-CoV (24), by immunostaining. Additional RT-qPCR assays detected transcripts for *ACE2* and *TMPRSS2* predominantly in hLO and hLO ALI (Fig. 2H), with higher transcript levels in hLO ALI, which is consistent with our immunostaining findings. Meanwhile, we also detected *DPP4* transcripts in hLO and hLO ALI, but not in hLOm (Fig. 2H). These results suggest that in addition to differences in cellular composition and architecture, hLO cells under different culture formats may differ in their surface proteins, such as viral receptor expression profiles and, thus, susceptibility to coronavirus infection.

### 2D and 3D culture formats influence SARS-CoV-2 infectivity

**Fig 3:**
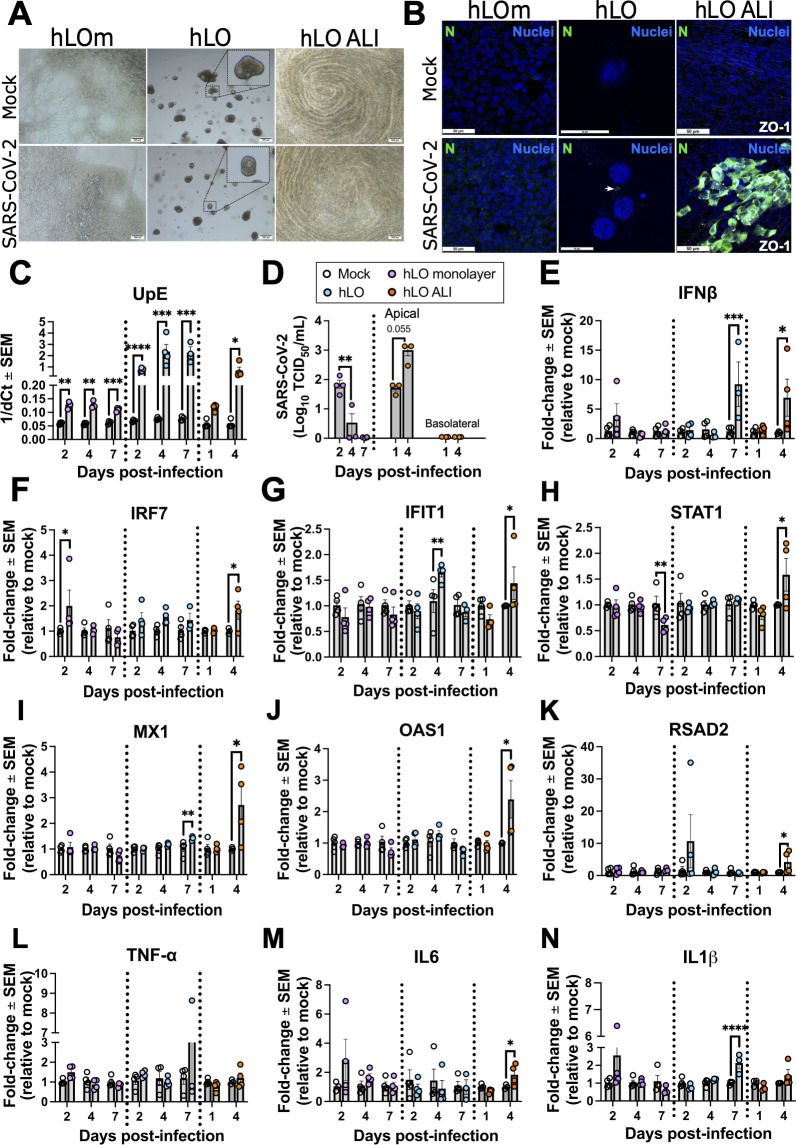
SARS-CoV-2 infectivity in donor-derived lung organoids (hLO), organoid-derived monolayers (hLOm), and ALI (hLO ALI) cultures. (**A**) Brightfield (5×) and (**B**) confocal imaging of immunostained cells mock infected or infected with SARS-CoV-2 at an MOI of 0.3 at 4 days post infection. Cells were fixed, permeabilized, and stained with antibodies against the SARS-CoV-2 nucleoprotein (**N**) and DAPI (nuclei) and imaged at 63×. Scale bars correspond to 200 µm (**A**) and 50 µm (**B**). (**C**) Total RNA was collected at specified times to determine expression of a viral genomic region upstream the E gene (UpE). (**D**) SARS-CoV-2 virus titers in supernatant of monolayers, and apical and basolateral media in ALI cultures assessed using TCID_50_ assay. Host gene expression of *IFNβ* (**E**), *IRF7* (**F**), *IFIT1* (**G**), *STAT1* (**H**), *MX1* (**I**), *OAS1* (**J**), *RSAD2* (**K**), *TNF-α* (**L**), *IL6* (**M**), and *IL1β* (**N**) was evaluated by RT-qPCR. For RT-qPCR assays, samples were assayed in technical duplicates, dCt was normalized to GAPDH, and virus gene expression was calculated as 1/dCt. Host gene expression was calculated as fold-change relative to the mean of mock controls by 2^−ddCt^ method. The mean of three independent samples is presented; error bars are standard error of the mean (SEM). Empty circles = mock; Purple circles = hLOm; Teal circles = hLO; Orange circles = hLO ALI. Statistical analysis was performed by one-way ANOVA (**C–M**) or Student’s *t* test (**N**). *, *P* < 0.05; **, *P* < 0.01; ***, *P* < 0.001; ****, *P* < 0.0001.

Since 2D and 3D cultures differed in cell composition, architecture, and breadth of response against pathogen-like stimuli, we next investigated whether culture format also impacts infection with respiratory viruses. We first used SARS-CoV-2 due to its recent relevance for global health. Parental hLO, hLOm, and hLO ALI cultures were infected with SARS-CoV-2 and followed for up to 7 days. Brightfield images suggested that hLO monolayers were composed of cells with epithelial-like morphology (Fig. 3A). Meanwhile, hLOs retained characteristic spherical morphology with presence of cyst-like cavities and absence of cilia on the apical aspect of the organoids (apical-in). hLO ALI displayed airway-like characteristics with beating cilia that actively moved mucus atop the culture, giving the appearance of whorls when viewed from top down (Fig. 3A).

Despite the absence of apparent cytopathic effect (CPE) in all three types of culture upon SARS-CoV-2 infection (Fig. 3A), immunofluorescence staining confirmed the presence of the SARS-CoV-2 N protein in hLO and hLO ALI cultures (Fig. 3B). We evaluated tight junction status in ALI cultures by staining for the ZO-1 marker (Fig. 3B), which appeared unaffected by SARS-CoV-2 infection in our experimental conditions (Fig. 3B). We did not detect cells positive for SARS-CoV-2 N protein in hLOm (Fig. 3B). These observations were further confirmed by RT-qPCR assays which showed higher upstream of E gene (UpE) transcripts (19) in hLO and hLO ALI cultures upon infection with SARS-CoV-2 when compared to hLOm (Fig. 3C).

We detected SARS-CoV-2 transcripts in hLOm (low), hLO, and hLO ALI (UpE, Fig. 3C). However, we did not detect SARS-CoV-2 N positive cells in hLOm, whereas hLO ALI cultures infected with SARS-CoV-2 had several cells positive for N (Fig. 3B). We performed TCID_50_ assays to determine whether hLOm or hLO ALI produced infectious progeny virions given that virus UpE levels were lower in these formats relative to hLO (Fig. 3D). Infected hLO ALI produced increasing amounts of infectious virus at the apical side of the ALI. In hLOm, virus was detected 2 days post-infection which then decreased over 4- and 7-days post infection (Fig. 3D). We did not detect infectious virus in ALI basolateral medium (Fig. 3D).

We then profiled the immune responses that were generated in the three culture systems upon SARS-CoV-2 infection. hLO and ALI cultures upregulated *IFNβ* transcripts at 7- and 4-days post-infection, respectively (Fig. 3E). SARS-CoV-2 infection led to the upregulation of several canonical antiviral ISGs in hLO ALI including IFN regulatory factor 7 (*IRF7*) (Fig. 3F), *IFIT1* (Fig. 3G), *STAT1* (Fig. 3H), *MX1* (Fig. 3I), *OAS1* (Fig. 3J), and Radical S-Adenosyl Methionine Domain Containing 2 (*RSAD2*) (Fig. 3K) at 4 days post infection. In case of hLO, SARS-CoV-2 infection led to the upregulation of *IFIT1* (Fig. 3G), with the remaining ISGs failing to respond to virus infection. Analyses of key proinflammatory genes (Fig. 3L through N) demonstrated the upregulation of transcripts for Interleukin 6 (*IL6*) in hLO ALI at 4 dpi (Fig. 3M), whereas transcripts for *IL1β* were upregulated in hLO at 7 dpi (Fig. 3N). hLOm remained mostly unresponsive to virus infection and did not upregulate transcripts for our selected antiviral genes other than *IRF7* at 2 dpi (Fig. 3F). Thus, hLO ALI cultures were more responsive to SARS-CoV-2 infection, relative to parental hLO and hLOm. These results show that despite their common origin, culture format impacts SARS-CoV-2 infection kinetics and immune response in primary donor lung organoid cells.

### 2D and 3D culture formats impact MERS-CoV infectivity

**Fig 4:**
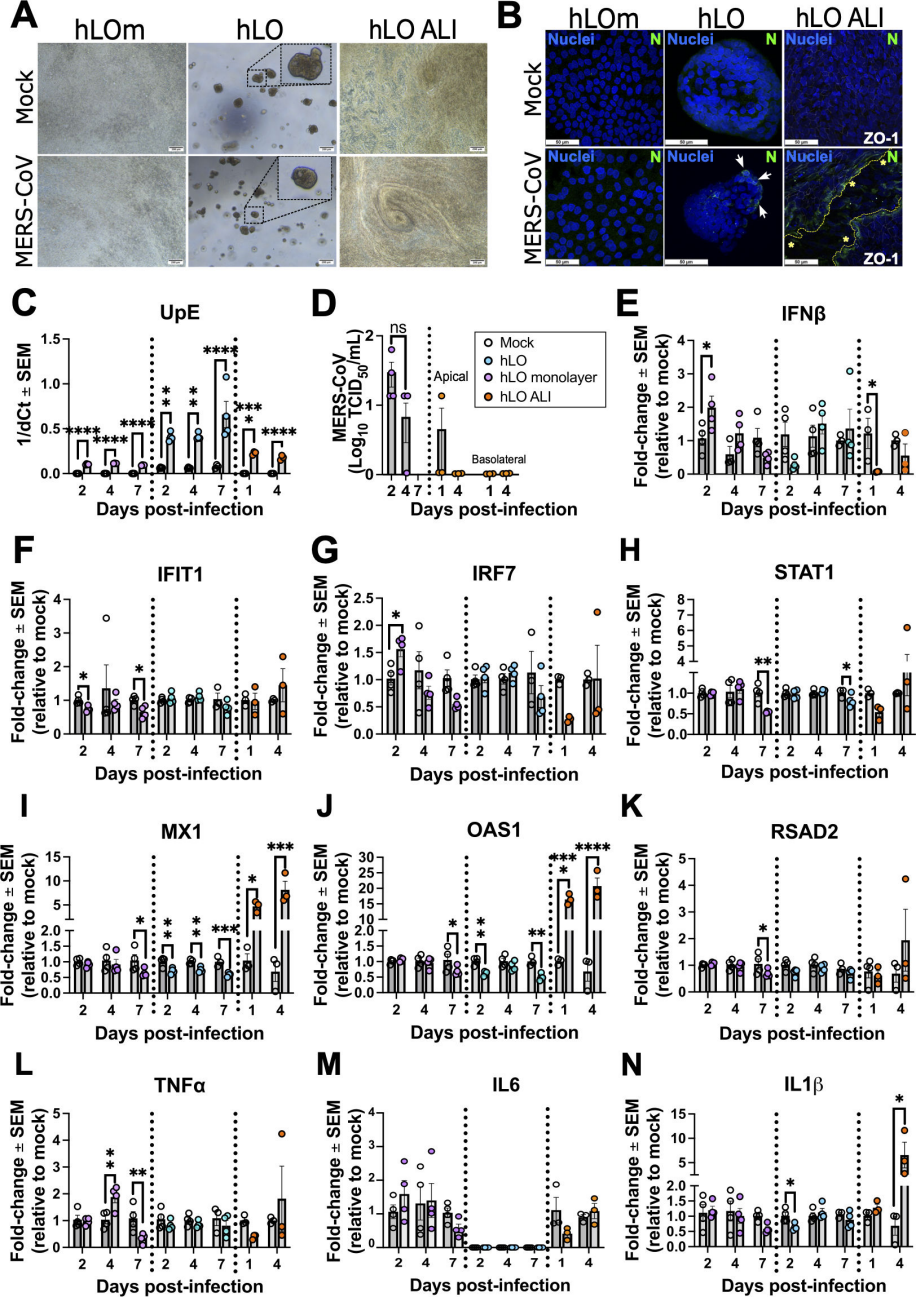
MERS-CoV infectivity in donor-derived lung organoids (hLO), organoid-derived monolayers (hLOm), and ALI (hLO ALI) cultures. (**A**) Brightfield and (**B**) confocal imaging of immunostained cells mock infected or infected with MERS-CoV at an MOI of 0.3 at 4 days post infection. Cells were fixed, permeabilized, and stained with antibodies against the MERS-CoV nucleoprotein (**N**) and DAPI (nuclei) and imaged at 63×. Scale bars correspond to 200 µm (**A**) and 50 µm (**B**). White arrows indicate infected cells and dashed lines and asterisks (yellow) delineate areas within the ALI in which ZO-1 was absent, and the culture integrity was disrupted (**B**). (**C**) Total RNA was collected at specified times to determine expression of a viral genomic region upstream the E gene (UpE). (**D**) MERS-CoV virus titers in supernatant of hLOm and apical and basolateral media of hLO ALI assessed using TCID_50_ assay. Host gene expression of *IFNβ* (**E**), *IRF7* (**F**), *IFIT1* (**G**), *STAT1* (**H**), *MX1* (**I**), *OAS1* (**J**), *RSAD2* (**K**), *TNF-α* (**L**), *IL6* (**M**), and *IL1β* (**N**) was evaluated by RT-qPCR. The mock infected samples for hLO ALI are the same as in Fig. 3. For RT-qPCR assays, samples were assayed in technical duplicates, dCt was normalized to GAPDH, and virus gene expression was calculated as 1/dCt. Host gene expression was calculated as fold-change relative to the mean of mock infected controls using the 2^−ddCt^ method. The mean of three independent samples is presented; error bars are standard error of the mean (SEM). Empty circles = mock; Purple circles = hLOm; Teal circles = hLO; Orange circles = hLO ALI. Statistical analysis was performed by one-way ANOVA (**C–M**) or Student’s *t* test (**N**). *, *P* < 0.05; **, *P* < 0.01; ***, *P* < 0.001; ****, *P* < 0.0001.

We next investigated whether culture format also impacts MERS-CoV infection kinetics and host response. Like SARS-CoV-2, we did not observe CPE under bright field microscopy in either hLOm, hLO, or hLO ALI infected with MERS-CoV (Fig. 4A). Both hLO and hLO ALI had cells that stained positive for MERS-CoV nucleoprotein (N) (Fig. 4B). ALI cultures infected with MERS-CoV had areas of cell detachment, leaving gaps lined by infected cells and exposed cells in the basement layer that appeared as plaque-like lesions (Fig. 4B). These disrupted areas had discontinuous distribution of the ZO-1 marker, suggesting loss of tight junction integrity (Fig. 4B). RT-qPCR assays confirmed upregulation of viral gene transcripts (UpE) (25) in MERS-CoV infected hLOm, hLO, and hLO ALI cultures although hLOm and hLO ALI cultures did not show time-dependent increase of UpE (Fig. 4C).

Next, we performed TCID_50_ assays to determine whether hLOm and hLO ALI cultures produced infectious progeny virions into culture media and the apical side of ALIs (Fig. 4D). Like SARS-CoV-2, hLOm produced infectious MERS-CoV at 2 dpi, which decreased over time (Fig. 4D). Infectious MERS-CoV in apical washes of hLO ALIs or in the basolateral medium remained low or undetectable (Fig. 4D). Transcripts for *IFNβ* (Fig. 4E) and *IRF7* (Fig. 4G) were upregulated at early time points post infection in hLOm. *IFIT1* transcript levels remained largely unchanged (Fig. 4F). Transcripts for antiviral genes like *MX1* (Fig. 4I) and *OAS1* (Fig. 4J), along with proinflammatory genes like *IL1β* (Fig. 4N), were upregulated following infection with MERS-CoV in hLO ALI. Transcript levels for *IFIT1*, *STAT1*, *RSAD2*, *TNFα*, and *IL6* remained largely unchanged or were minimally differentially regulated upon infection with MERS-CoV (Fig. 4F, H, K, L and M). In hLOs, we noted mostly discrete downregulation of antiviral and proinflammatory gene transcripts. These results show that hLO ALI partly recapitulated host responses against MERS-CoV infection, which were not detected in hLO and hLOm cultures.

## DISCUSSION

Lung organoids are emerging tools to identify and model respiratory infectious diseases due to their tissue-like cellular heterogeneity and three-dimensional organization. Here, we generated donor-derived lung organoids and used them to make two-dimensional cell monolayers (hLOm) and three-dimensional ALI (hLO ALI) cultures. We compared infection kinetics of SARS-CoV-2 and MERS-CoV and associated host responses in these three culture formats. Relative to hLOm and parental hLO, hLO ALI were more responsive to virus infection, allowing for the identification of immune response signatures corresponding to SARS-CoV-2 and MERS-CoV. Our comparative study shows that despite sharing a common origin and genetic background, each culture format had distinctive virus infection and host response patterns.

Although organoids do not reflect the full composition or functionality of the lungs, they can partially recapitulate key features of the respiratory tract to a better extent than traditional 2D cell lines (4). Organoid culture is achieved through growth in gel-like matrix supports to ensure survival and differentiation of stem cells into cell types typical of the tissue of origin. Growth in basement membrane matrix gel prevents exposure to air while promoting growth of organoids as multicellular aggregates typically in the apical-in orientation wherein functional cilia does not develop (26). Despite these disadvantages, organoids from PSC (10, 27), iPSCs, and adult stem cells (9) have been used to generate nasal (28), tracheobronchial ⁠ (27), and alveolar organoids (29) to model SARS-CoV-2 infection. Studies generating organoids from iPSCs (30), embryonic stem cells or ESCs (31), and adult lung tissue (10) have reported that the origin and culture conditions of stem cells can influence the cell population that are present in final organoids, along with differences in their immune competency (32), which, in turn, may influence which cells are infected with SARS-CoV-2. Therefore, donor-specific traits (32), origin of stem cells, and types of cells within organoids may shape the antiviral response against virus infections.

The use of hESCs and hPSCs may not be feasible for every virology lab as access to these cells and reagents can be expensive, and the extensive time required to culture organoids can be limiting. Given the recent drive from the FDA (US) to de-risk preclinical testing by embracing organoid models and their inherent donor-to-donor variation (5), we used adult lung tissue as a source for stem cells. Culture of organoids from adult lung tissue follows procedures that can be easily adopted by the wider virology community and requires equipment that is already available in most virology laboratories. In addition to SARS-CoV-2, our goal was to evaluate this platform for MERS-CoV since limited studies have used respiratory organoids for MERS-CoV research (33, 34).

Access to tissues from healthy adult donors may be limiting. Biobanks cataloging and storing patient-derived cancer tissue are available through academic (e.g., Princess Margaret Living Biobank, the Hubrecht Institute) and commercial vendors (e.g., Cellesce, DefiniGEN). Biobanks for healthy tissues are not well developed and require partnership with clinicians and other researchers. While partnerships with academic hospitals is the most frequent way to secure healthy tissue for organoid-based research, regulated commercial enterprises (e.g., HUB organoids) are making headway in bringing these services to academic users.

As reported previously, we found that organoids generated from healthy donor tissue differentiated into cell types typical of bronchial airway tissue in the apical-in orientation due to the use of matrigel (Fig. 1) and were readily infectable by SARS-CoV-2 (Fig. 3) and MERS-CoV (Fig. 4). However, infecting hLOs involved the dissolution of matrigel, subsequent recovery of matrix-free organoids followed by virus infection in suspension, and organoid re-embedding in matrix, which is a complex process that does not facilitate high-throughput work. While virus infection was successful in matrigel-free whole organoids by us in this study and by others (35), methods like mechanical shearing or microinjection are also used to facilitate infection of apical-in organoids ⁠ (36). Recently developed microinjection platforms can process ~90 organoids per hour (37), but require specialized equipment and lack high-throughput capabilities, which curtails microinjection use in higher containment level laboratories. Microinjection platforms can be considered for infection of organoids; however, these protocols will need to be carefully assessed by institutional biosafety committees for biosafety risks before they can be implemented.

Given current challenges, we were prompted to explore the versatility of organoids in two additional culture formats: as cell monolayers (2D) and as ALI cultures (3D). This approach examines the intra-individual differences in organoid culture under the same genetic background, facilitating comparison between organoid culture formats to inform on choice criteria for virology research. Cell line monolayers composed of homogenous cell populations expressing virus receptors are the cornerstone for studies on SARS-CoV-2 and MERS-CoV. Cell lines like Calu-3 (38, 39), Huh-7 (40), MRC-5 ⁠ (41), and the non-human primate cell line Vero and its derivates ⁠ (42) have been widely used in coronavirus biology research. We used organoids to produce monolayers and analyzed virus infection kinetics. Unlike cell lines in which viruses cause widespread damage (cytopathic effect) due to quick and efficient replication, monolayers derived from primary organoids were infected poorly by SARS-CoV-2 (Fig. 3) and MERS-CoV (Fig. 4). Transition from organoids into monolayers led to the loss of detectable ACE2 and TMPRSS2 expression levels (Fig. 2H), resulting in a loss of viral infectivity in these cells.

hLOm were found to be immunocompetent against viral dsRNA analogs (Fig. 2A through F) but were unresponsive to bacterial LPS (Fig. 2G), indicating that monolayers do not retain the full breadth of immune response signaling. Human primary tracheobronchial cells upregulate IL6 and IL-1β when exposed to high doses (10–100 µg/mL) of LPS *in vitro* (43) although high LPS dosage has also been linked to loss of viability and subsequent release of inflammatory markers (44). However, features observed in primary cell cultures cannot be extended to hLOm due to differences in culture establishment and conditions such as growth media. In our study, the addition of a single growth factor (Epidermal Growth Factor, EGF) was required to direct monolayer-like growth from dissociated organoid cells. In epithelial cells, LPS engages TLR4 at the cell surface in the canonical pathway, which results in transactivation of the EGF receptor (EGFR) with subsequent NF-κβ activation in LPS-dependent acute lung injury models (45). While additional studies are needed to clarify the mechanisms by which hLOm lose responsiveness to LPS, we surmise that it is possible that EGF occupancy or regulation of EGFR may interfere with LPS-dependent inflammation.

Our data suggest that cell monolayers derived from organoids may not be appropriate models for mechanistic studies on pathogen-host interactions and care must be observed about the utility of these models to study antiviral response. In contrast, hLO ALI cultures could be infected with SARS-CoV-2 which led to the production of infectious progeny virions that were released at the apical side of the ALI membrane (Fig. 3). Our studies adopted a multi-pronged approach of immunofluorescence, RT-qPCR, and TCID50 assays to determine virus infection. These assays vary in their sensitivity and may present discrepancies that justify their combined use for evaluating virus infections. RT-qPCR detects low levels of transcripts, whereas immunofluorescence requires higher levels of viral proteins for visualization using microscopy. An additional hurdle arises in TCID50 assays to detect viruses in culture supernatant from matrigel-embedded organoids. Some groups have reported that viruses become entrapped in matrigel and are difficult to recover from medium (29, 46), whereas other groups report no issues in virus recovery from matrigel supernatants (47). Given these conflicting reports, additional steps may be needed to recover matrigel-entrapped viruses in high-containment facilities, along with complementary quantification of virus infection by immunofluorescence and RT-qPCR. We did not attempt TCID50 assays using culture supernatant from hLOs embedded in matrigel.

ALI cultures upregulated a diverse array of antiviral transcripts more frequently than parental organoids (Fig. 3E through N). Our study is consistent with previous work highlighting the improved immune response against SARS-CoV-2 in ALI cultures, including those generated from primary bronchial cells (48). The commercialization of transwell inserts and ready-made cell culture reagents have facilitated the establishment of ALI cultures from different cell sources with minimal modifications. Indeed, commercial vendors (e.g., StemCell, Lonza, Corning) have made significant advances in pre-made media and plasticware for 3D culture, facilitating its adoption across disciplines.

Studies exploring MERS-CoV infection in organoids and ALI cultures are scarce. We investigated both culture formats and found that parental organoids (hLO) and hLO ALI were permissive to MERS-CoV infection (Fig. 4). Our findings are consistent with a recent report in human airway organoids suggesting that merbecoviruses likely infect airway organoids with low efficiency (49). Responses against MERS-CoV were centered on upregulation of antiviral transcripts for *MX1* (Fig. 4I) and *OAS1* (Fig. 4J) as reported in other *in vitro* studies (50, 51). Interestingly, *MX1* and *OAS1* transcripts were downregulated in parental organoids and hLOm infected with MERS-CoV. Induction of *IFNβ* transcripts was largely absent in hLO and hLO ALI cultures, demonstrating that 3D models can also emulate this aspect of MERS-CoV IFN antagonism (52). MERS-CoV infection induced the upregulation of transcripts for proinflammatory cytokines like *IL-1β* in hLO ALI cultures, but not in the other formats (Fig. 4N). Thus, in our studies, hLO ALI models better recapitulated responses that underlie hyperinflammatory syndromes in patients with severe MERS (53) and virus-mediated IFN antagonism.

MERS-CoV induces CPE or cell death in various human and non-human cell lines like Calu3, Huh7, Vero, and derivates (42, 54). MERS can induce sloughing of infected cells in ALI cultures made from primary human tracheobronchial cells (55). Instead of sloughing, we identified dissolution of tight junctions at the epithelial barrier of organoid-derived ALIs where plaque-like lesions lined by infected cells had formed (Fig. 4B). Disturbances in the epithelial barrier have also been identified in human alveolar tissue infected with MERS-CoV *ex vivo* (56) and in postmortem histopathological exams (57). Our study suggests that organoids at the ALI can recapitulate part of the virus-induced tissue damage and immune responses better than their parental organoids. Neither SARS-CoV-2 nor MERS-CoV induced extensive CPE reported in 2D cell lines although cell lines do not necessarily reflect the diverse pathologies observed in infected individuals. Cell lines are homogeneous in their expression of virus receptors and support of virus replication, which intensifies the appearance of CPE. On the contrary, cellular and phenotypic heterogeneity in the lungs occurs at the intra-individual level, thus governing different populations of cells that are affected at any given time in an individual. In this fundamental difference, 3D models partly capture the general heterogeneity and intrinsic variability of the lungs better than traditional cell cultures.

A potential drawback of our approach is the use of tissue obtained from a single donor to generate organoids and organoid-derived cultures. Therefore, while addressing intra-donor variation across culture formats, our study does not address donor-to-donor differences that influence susceptibility and host responses against virus infections. Despite this limitation, and in the background of a shared origin and genetic makeup, 2D and 3D cell culture formats influenced pathogen kinetics and host immune responses in our studies. These differences between 2D and 3D cultures must be taken into consideration when selecting models to investigate respiratory diseases. As technology continues to advance, bioengineering approaches like microfluidic systems can help refine current respiratory models for infectious disease research and therapeutic testing. Indeed, our work informs the selection and use of human-derived models compatible with biomimetic approaches for infectious disease research and therapeutic screening. Given the limitations of organoids that typically grow in apical-in orientations, organoids at the ALI are a more reliable and reproducible model for infection and immune response studies that partially mimic the air-liquid environment of the lungs.

## MATERIALS AND METHODS

### Generation of human lung organoids

Human lung organoids (hLO) were established as described by Sachs (15) from a healthy tissue sample obtained from the upper lobe of a male patient undergoing surgery. Briefly, lung tissue was minced in Advanced DMEM/F-12 (Gibco, cat. 12634010) before single cell dissociation in ACF dissociation solution (StemCell, cat. 05426) for 1.5 h at 37°C. Undigested tissue was pelleted, and the cell suspension was strained through a 37 µm mesh reversible strainer. Red blood cells were lysed with ACK lysing buffer (Gibco, cat. A1049201), and the remaining cells were embedded in Matrigel (Corning, cat. CACB356231). Matrigel-embedded cells (P0) were dispensed as 40 µL dome/well on three wells of a pre-warmed 12-well tissue culture plate, left to solidify at room temperature for 2 min and then incubated upside down at 37°C for 10 min. Solidified domes were submerged in 400 µL of complete organoid media ⁠(15) (Table 1) supplemented with antibiotic-antimycotic (Gibco, cat. 15240096), with media changes every 3–4 days. hLO were passaged (1:6 ratio) every 7–10 days by dissociation at 37°C (TrypLE Express 1X) into clusters of 3–4 cells each. Cell clusters were washed in advanced DMEM, re-embedded into fresh Matrigel, and dispensed as 25 µL dome/well of a pre-warmed 24-well tissue culture plate, left to solidify as before and submerged in 250 µL of complete organoid media. To generate cell monolayers (hLOm), hLOs were dissociated into clusters of 3–4 cells (TrypLE Express), washed, and seeded in pre-warmed cell culture plates using complete hLO media supplemented with EGF (20 ng/mL). Media was changed every 3–4 days until reaching 80% confluence before passage or used in indicated experiments. Air-liquid interface cultures (hLO-ALI) were generated using hLOs dissociated as before and seeded at ~1–3 × 10^5^ cells/200 µL in hLO complete media supplemented with EGF (20 ng/mL) on the top chambers of 12 mm transwell inserts (StemCell, cat. 38024). Bottom chambers were filled with 500 µL of hLO complete media supplemented with EGF, and cell-laden transwells were incubated at 37°C for 2–4 days. The top chamber was exposed to the air interface for 28 days to allow for bronchiolar airway-like differentiation, and bottom chamber media was replaced with ALI maintenance medium (Pneumacult-ALI, StemCell, cat. 05001) and changed every 2 days. The apical aspect of ALI cultures was washed in 200 µL of PBS for 10 min at 37°C at least once a week after exposure to air to remove mucus buildup.

**TABLE 1:** Complete organoid media (15)

Component	Catalog number	Manufacturer	Final concentration
R-Spondin 1	120-38	Peprotech	500 ng/mL
FGF7	100-19	Peprotech	25 ng/mL
FGF10	100-26	Peprotech	100 ng/mL
Noggin	120-10C	Peprotech	100 ng/mL
A83-01	2939	Tocris	500 nM
Y-27632	Y-27632	StemCell	5 µM
SB202190	S7067	Sigma	500 nM
B27	17504-44	Gibco	1×
N-Acetylcysteine	A9165-5g	Sigma	1.25 mM
Nicotinamide	N0636	Sigma	5 mM
Glutamax 100×	12634-034	Invitrogen	1×
HEPES	15630-056	Invitrogen	10 mM
Anti-anti 100×	15240062	Gibco	1×
Advanced DMEM/F12	12634-034	Invitrogen	1×

### Virus infection

SARS-CoV-2/SB2 clinical isolate ⁠ (58) and MERS-CoV isolate EMC/2012 were used in these experiments. hLOs were released from Matrigel in ice cold gentle harvesting buffer (Cultrex, cat. 3700-100-01) and infected at an MOI of 20 per hLO (~0.3 per cell) for 2 h at 37°C in Advanced DMEM/F-12. hLOs were washed twice to remove unbound virus, re-embedded in Matrigel, and cultured in hLO complete media for indicated times. Monolayers were cultured until 80% confluence, washed with PBS, and infected at MOI 0.3 for 1 h at 37°C. Cells were PBS washed and incubated for the indicated times in hLO complete media supplemented with EGF. The apical side of differentiated ALI cultures was washed with PBS pre-warmed at 37°C for 10 min to remove mucus prior to infection. ALIs were infected at MOI 0.3 for 1 h at 37°C to mimic virus entry through the air interface. Infected ALIs were washed twice and incubated in ALI maintenance medium for indicated times. After indicated times, total RNA was collected using the RNeasy Mini kit (Qiagen, cat 74104) following the manufacturer’s instructions. Work with infectious virus was performed in containment level 3 (CL-3) facilities at VIDO-Intervac, University of Saskatchewan.

### Immunocompetence assays

hLOs, hLOm, and hLO-ALI were exposed to LPS 100 ng/mL (Invivogen, cat tlrl-eblps) prepared in complete media for 6 h. For transfection assays, organoids were recovered from matrigel using ice cold gentle harvesting buffer (Cultrex, cat. 3700-100-01) and transfected in suspension with poly(I:C) rhodamine (InvivoGen, cat tlrl-picr) at increasing concentrations for 6 h using lipofectamine 3000 (Invitrogen, cat. L3000015). Transfected organoids were then re-embedded in fresh matrigel and incubated for 48 h. RNA was extracted with the RNeasy Mini kit (Qiagen, cat 74104) following the manufacturer’s instructions.

### RT-qPCR

cDNA synthesis from ~500 ng of RNA was generated with the iScript gDNA Clear cDNA Synthesis Kit following the manufacturer’s instructions (BioRad 1725034). cDNA was diluted 1:10 in RNAse free water and used as template for qPCRs using Ssoadvanced Universal SYBR kit following the manufacturer’s instructions (BioRad 1725274) using selected primers (Table 2). UpE primers for SARS-CoV-2 and MERS-CoV were utilized to detect both genomic and subgenomic RNA from actively replicating virus (19, 25). For RT-qPCR assays, samples were assayed in technical duplicates, dCt was normalized to GAPDH, and virus gene expression was calculated as 1/dCt. qPCRs were performed in a StepOne Real Time PCR System (Applied Biosciences). We normalized Ct values by GAPDH (dCt) and used 1/dCt for viral transcript levels (UpE), and 2^−ddCt^ method for quantitation of host gene expression relative to time-matched mocks or vehicle controls.

**TABLE 2:** Primers used in this study to determine expression of host and virus genes

Primer name	Sequence (5′–3′)
IRF7 Fw	CCCACGCTATACCATCTACCT
IRF7 Rv	GATGTCGTCATAGAGGCTGTTG
STAT1 Fw	GTTGGTGAAATTGCAAGAGCTG
STAT1 Rv	GGTGGACTCCTCCATGTTCATC
IFNβ Fw	GCTTGGATTCCTACAAAGAAGCA
IFNβ Rv	ATAGATGGTCAATGCGGCGTC
IFIT1 Fw	GGCAGAAGCCCAGACTTACC
IFIT1 Rv	GGGTCCACTTCAAGCACCTT
OAS1 Fw	AGT TGA CTG GCG GCT ATA AAC
OAS1 Rv	GTG CTT GAC TAG GCG GAT GAG
MX1 Fw	AGG ACT ACG AGA TTG AGA T
MX1 Rv	TTA TGC CAG GAA GGT CTA
IL1 Fw	ATGATGGCTTATTACAGTGGCAA
IL1 Rv	GTCGGAGATTCGTAGCTGGA
TNFα Fw	CAGCCTCTTCTCCTTCCT GA
TNFα Rv	AGATGATCTGACTGCCT GGG
IL6 Fw	ACTCACCTCTTCAGAACGAATTG
IL6 Rv	CCATCTTTGGAAGGTTCAGGTTG
RSAD2 Fw	TGGGTGCTTACACCTGCTG
RSAD2 Rv	GAAGTGATAGTTGACGCTGGTT
SCGB1A1 Fw	TTCAGCGTGTCATCGAAACCC
SCGB1A1 Rv	ACAGTGAGCTTTGGGCTATTTTT
FoxJ1 Fw	GTGCTTCATCAAAGTGCCTCG
FoxJ1 Rv	GCCTCGGTATTCACCGTCA
TUBB4B Fw	GGACAACTTCGTTTTCGGTCA
TUBB4B Rv	CCTTTCTCACAACATCCAGCAC
‍TJP1 Fw	ACCAGTAAGTCGTCCTGATCC
‍TJP1 Rv	TCGGCCAAATCTTCTCACTCC
‍TP63 Fw	GGACCAGCAGATTCAGAACGG
‍TP63 Rv	AGGACACGTCGAAACTGTGC
KRT5 Fw	AGGAGTTGGACCAGTCAACAT
KRT5 Rv	TGGAGTAGTAGCTTCCACTGC
‍NGRF Fw	TGGCCTACATAGCCTTCAAGA
‍‍NGRF Rv	GAGATGCCACTGTCGCTGT
Muc5AC Fw	CAGCACAACCCCTGTTTCAAA
Muc5AC Rv	GCGCACAGAGGATGACAGT
‍PDPN1 Fw	GTGTAACAGGCATTCGCATCG
‍‍PDPN1 Rv	TGTGGCGCTTGGACTTTGT
AQP5 Fw	CGGGCTTTCTTCTACGTGG
AQP5 Rv	GCTGGAAGGTCAGAATCAGCTC
‍SFTPC Fw	CACCTGAAACGCCTTCTTATCG
‍‍SFTPC Rv	TTTCTGGCTCATGTGGAGACC
SLC34A2 Fw	CTGAGGCACCTGTAACCAAGA
SLC34A2 Rv	TGATCCCCGAGTCCTGAAGAG
SARS-CoV-2 UpE Fw (19)	ATTGTTGATGAGCCTGAAG
SARS-CoV-2 UpE Rw (19)	TTCGTACTCATCAGCTTG
MERS-CoV UpE Fw (25)	GCAACGCGCGATTCAGTT
MERS-CoV UpE Rw (25)	GCCTCTACACGGGACCCATA

### Immunocytochemistry

Matrigel-free hLOs fixed in neutral buffered formalin (NBF) 10% were blocked and permeabilized for 2 h at room temperature (5% BSA, 1% Triton-X-100, 0.1% Tween-20 in PBS). Incubation with primary antibodies was performed at 1:250 dilution (Table 3) in IF buffer (0.1% BSA, 0.2% Triton-X-100 in PBS) overnight at 4°C. Samples were washed in IF buffer thrice before addition of labeled secondary antibodies (1:3,000). Nuclei were counterstained with DAPI (4 µg/mL) prepared in IF buffer, rinsed once in PBS and once in distilled water, and mounted on glass slides using ProLong Gold antifade mountant (Invitrogen, cat. P36930). Monolayers cultured in chambered slides (IBIDI 80806) were fixed with NBF 10% and processed as in hLOs. hLO ALI samples were fixed and cryoprotected in 30% sucrose for 24 h, embedded in Tissue TEK- O.C.T compound (Sakura), and frozen in cryomolds at −20°C. Transections of 4 µm were prepared by cryo-sectioning and processed for labeling as in hLOs. Samples were imaged in Leica TCS SP8.

**TABLE 3:** Antibodies used in this study

Antibody	Company	Catalog number
TMPRSS2 (H-4) AF546	Santa Cruz Biotechnology	sc-515727
ACE2	Invitrogen	MA5-41038
MUC5AC (45M1) AF647	Santa Cruz Biotechnology	sc-21701
ZO-1 AF647	Invitrogen	MA3-39100-A647
CC10 (E-11) AF546	Santa Cruz Biotechnology	sc-365992
KRT5 (RCK103) AF488	Santa Cruz Biotechnology	sd-32721
Podoplanin PDPN1 (E-1) AF647	Santa Cruz Biotechnology	SC-376695
Surfactant protein SP-C (H-8) AF594	Santa Cruz Biotechnology	SC-518029
Acetylated tubulin (6-11B-1) AF594	Santa Cruz Biotechnology	SC-23950
SARS-CoV-2 nucleoprotein N	Invitrogen	MA5-29981
MERS-CoV nucleoprotein N	Sino Biological	40068-RP01-200
Goat anti-mouse IgG (H + L) highly-crossed adsorbed secondary antibody, AF647	Invitrogen	A32728
Goat anti-rabbit IgG (H + L) highly-crossed adsorbed secondary antibody, AF488	Invitrogen	A32731TR
Goat anti-mouse IgG (H + L) Superclonal secondary antibody, AF488	Invitrogen	A28175
Goat anti-rabbit IgG (H + L) highly-crossed adsorbed secondary antibody, AF594	Invitrogen	A32740

### Virus titration (TCID_50_ assays)

Apical washes and basal medium of mock and infected ALI cultures were collected for titration assays. Apical washes were obtained by adding 200 µL of PBS to the apical side of ALI cultures and incubating for 10 min at room temperature before collection. Supernatant from cell monolayers were also collected. Virus titration was performed as described previously (19). In brief, Vero76 cells were seeded at 2 × 10^4^ cells per well in 96 well plates and incubated for 24 h at 37°C in complete media (DMEM supplemented with 10% FBS, 1% penicillin-streptomycin, and 1× Glutamax). Vero 76 cells were infected in triplicates with undiluted and tenfold diluted samples prepared in serum free DMEM for 1 h at 37°C. Inoculum was removed, and Vero76 cells were reconstituted with 2% FBS media and incubated at 37°C (5% CO_2_) for up to 5 days. Cells were monitored at 3- and 5-days post infection to determine CPE. Virus titers were calculated according to the Spearman and Karber method. The lower limit of detection for SARS-CoV-2 was 2.94 × 10^1^ TCID_50_/mL and for MERS-CoV it was 1.36 × 10^1^ TCID_50_/mL in our assays.

### Statistics

Statistical analyses were performed in GraphPad Prism (version 10.1.0). RT-qPCR and TCID_50_ data were analyzed by one-way ANOVA or Student’s *t* test. Significance values and statistical tests are indicated in the figures and figure legends. *, *P* < 0.05; **, *P* < 0.01; ***, *P* < 0.001; and ****, *P* < 0.0001.

## Data Availability

All data generated and analyzed that support the findings of this study are included in the published article.

## References

[1] Pires De Souza GA, Le Bideau M, Boschi C, Wurtz N, Colson P, Aherfi S, Devaux C, La Scola B. 2022. Choosing a cellular model to study SARS-CoV-2. Front Cell Infect Microbiol 12:1003608. doi:10.3389/fcimb.2022.1003608 36339347 PMC9634005

[2] Rong N, Liu J. 2023. Development of animal models for emerging infectious diseases by breaking the barrier of species susceptibility to human pathogens. Emerg Microbes Infect 12:2178242. doi:10.1080/22221751.2023.2178242 36748729 PMC9970229

[3] Zhao Z, Chen X, Dowbaj AM, Sljukic A, Bratlie K, Lin L, Fong ELS, Balachander GM, Chen Z, Soragni A, Huch M, Zeng YA, Wang Q, Yu H. 2022. Organoids. Nat Rev Methods Primers 2:1–21. doi:10.1038/s43586-022-00174-y PMC10270325 37325195

[4] Kim J, Koo BK, Knoblich JA. 2020. Human organoids: model systems for human biology and medicine. Nat Rev Mol Cell Biol 21:571–584. doi:10.1038/s41580-020-0259-3 32636524 PMC7339799

[5] Han JJ. 2023. FDA modernization act 2.0 allows for alternatives to animal testing. Artif Organs 47:449–450. doi:10.1111/aor.14503 36762462

[6] Hui KPY, Ching RHH, Chan SKH, Nicholls JM, Sachs N, Clevers H, Peiris JSM, Chan MCW. 2018. Tropism, replication competence, and innate immune responses of influenza virus: an analysis of human airway organoids and ex-vivo bronchus cultures. Lancet Respir Med 6:846–854. doi:10.1016/S2213-2600(18)30236-4 30001996

[7] Harford TJ, Rezaee F, Dye BR, Fan J, Spence JR, Piedimonte G. 2022. RSV-induced changes in a 3-dimensional organoid model of human fetal lungs. PLoS One 17:e0265094. doi:10.1371/journal.pone.0265094 35263387 PMC8906588

[8] Zhao S, Wu X, Tan Z, Ren Y, Li L, Ou J, Lin Y, Song H, Feng L, Seto D, Wu J, Zhang Q, Rong Z. 2023. Generation of human embryonic stem cell-derived lung organoids for modeling infection and replication differences between human adenovirus types 3 and 55 and evaluating potential antiviral drugs. J Virol 97:e0020923. doi:10.1128/jvi.00209-23 37120831 PMC10231139

[9] Salahudeen AA, Choi SS, Rustagi A, Zhu J, van Unen V, de la O SM, Flynn RA, Margalef-Català M, Santos AJM, Ju J, et al.. 2020. Progenitor identification and SARS-CoV-2 infection in human distal lung organoids. Nature 588:670–675. doi:10.1038/s41586-020-3014-1 33238290 PMC8003326

[10] Lamers MM, van der Vaart J, Knoops K, Riesebosch S, Breugem TI, Mykytyn AZ, Beumer J, Schipper D, Bezstarosti K, Koopman CD, Groen N, Ravelli RBG, Duimel HQ, Demmers JAA, Verjans GMGM, Koopmans MPG, Muraro MJ, Peters PJ, Clevers H, Haagmans BL. 2021. An organoid-derived bronchioalveolar model for SARS-CoV-2 infection of human alveolar type II-like cells. EMBO J 40:e105912. doi:10.15252/embj.2020105912 33283287 PMC7883112

[11] COVID-19 deaths | WHO COVID-19 dashboard. 2023. World Health Organization

[12] Johns Hopkins University. 2022. Mortality analyses. Johns Hopkins Coronavirus Resource Center.

[13] Wang Q, Iketani S, Li Z, Liu L, Guo Y, Huang Y, Bowen AD, Liu M, Wang M, Yu J, Valdez R, Lauring AS, Sheng Z, Wang HH, Gordon A, Liu L, Ho DD. 2023. Alarming antibody evasion properties of rising SARS-CoV-2 BQ and XBB subvariants. Cell 186:279–286. doi:10.1016/j.cell.2022.12.018 36580913 PMC9747694

[14] Milne-Price S, Miazgowicz KL, Munster VJ. 2014. The emergence of the Middle East respiratory syndrome coronavirus. Pathog Dis 71:121–136. doi:10.1111/2049-632X.12166 24585737 PMC4106996

[15] Sachs N, Papaspyropoulos A, Zomer-van Ommen DD, Heo I, Böttinger L, Klay D, Weeber F, Huelsz-Prince G, Iakobachvili N, Amatngalim GD, et al.. 2019. Long-term expanding human airway organoids for disease modeling. EMBO J 38:e100300. doi:10.15252/embj.2018100300 30643021 PMC6376275

[16] Kesimer M. 2022. Mucins MUC5AC and MUC5B in the airways: MUCing around together. Am J Respir Crit Care Med 206:1055–1057. doi:10.1164/rccm.202208-1459ED 35938865 PMC9704829

[17] Blackburn JB, Li NF, Bartlett NW, Richmond BW. 2023. An update in club cell biology and its potential relevance to chronic obstructive pulmonary disease. Am J Physiol Lung Cell Mol Physiol 324:L652–L665. doi:10.1152/ajplung.00192.2022 36942863 PMC10110710

[18] Prescott RA, Pankow AP, de Vries M, Crosse KM, Patel RS, Alu M, Loomis C, Torres V, Koralov S, Ivanova E, Dittmann M, Rosenberg BR. 2023. A comparative study of in vitro air-liquid interface culture models of the human airway epithelium evaluating cellular heterogeneity and gene expression at single cell resolution. Respir Res 24:213. doi:10.1186/s12931-023-02514-2 37635251 PMC10464153

[19] Banerjee A, El-Sayes N, Budylowski P, Jacob RA, Richard D, Maan H, Aguiar JA, Demian WL, Baid K, D’Agostino MR, et al.. 2021. Experimental and natural evidence of SARS-CoV-2-infection-induced activation of type I interferon responses. iScience 24:102477. doi:10.1016/j.isci.2021.102477 33937724 PMC8074517

[20] Blanco-Melo D, Nilsson-Payant BE, Liu W-C, Uhl S, Hoagland D, Møller R, Jordan TX, Oishi K, Panis M, Sachs D, Wang TT, Schwartz RE, Lim JK, Albrecht RA, tenOever BR. 2020. Imbalanced host response to SARS-CoV-2 drives development of COVID-19. Cell 181:1036–1045. doi:10.1016/j.cell.2020.04.026 32416070 PMC7227586

[21] Jeyaseelan S, Chu HW, Young SK, Worthen GS. 2004. Transcriptional profiling of lipopolysaccharide-induced acute lung injury. Infect Immun 72:7247–7256. doi:10.1128/IAI.72.12.7247-7256.2004 15557650 PMC529166

[22] Hoffmann M, Kleine-Weber H, Schroeder S, Krüger N, Herrler T, Erichsen S, Schiergens TS, Herrler G, Wu NH, Nitsche A, Müller MA, Drosten C, Pöhlmann S. 2020. SARS-CoV-2 cell entry depends on ACE2 and TMPRSS2 and is blocked by a clinically proven protease inhibitor. Cell 181:271–280. doi:10.1016/j.cell.2020.02.052 32142651 PMC7102627

[23] Wu C-T, Lidsky PV, Xiao Y, Cheng R, Lee IT, Nakayama T, Jiang S, He W, Demeter J, Knight MG, Turn RE, Rojas-Hernandez LS, Ye C, Chiem K, Shon J, Martinez-Sobrido L, Bertozzi CR, Nolan GP, Nayak JV, Milla C, Andino R, Jackson PK. 2023. SARS-CoV-2 replication in airway epithelia requires motile cilia and microvillar reprogramming. Cell 186:112–130. doi:10.1016/j.cell.2022.11.030 36580912 PMC9715480

[24] Wang N, Shi X, Jiang L, Zhang S, Wang D, Tong P, Guo D, Fu L, Cui Y, Liu X, Arledge KC, Chen YH, Zhang L, Wang X. 2013. Structure of MERS-CoV spike receptor-binding domain complexed with human receptor DPP4. Cell Res 23:986–993. doi:10.1038/cr.2013.92 23835475 PMC3731569

[25] Corman VM, Eckerle I, Bleicker T, Zaki A, Landt O, Eschbach-Bludau M, van Boheemen S, Gopal R, Ballhause M, Bestebroer TM, Muth D, Müller MA, Drexler JF, Zambon M, Osterhaus AD, Fouchier RM, Drosten C. 2012. Detection of a novel human coronavirus by real-time reverse-transcription polymerase chain reaction. Euro Surveill 17:20285. doi:10.2807/ese.17.39.20285-en 23041020

[26] Kozlowski MT, Crook CJ, Ku HT. 2021. Towards organoid culture without matrigel. Commun Biol 4:1387. doi:10.1038/s42003-021-02910-8 34893703 PMC8664924

[27] Duan X, Tang X, Nair MS, Zhang T, Qiu Y, Zhang W, Wang P, Huang Y, Xiang J, Wang H, Schwartz RE, Ho DD, Evans T, Chen S. 2021. An airway organoid-based screen identifies a role for the HIF1α-glycolysis axis in SARS-CoV-2 infection. Cell Rep 37:109920. doi:10.1016/j.celrep.2021.109920 34731648 PMC8516798

[28] Li C, Huang J, Yu Y, Wan Z, Chiu MC, Liu X, Zhang S, Cai J-P, Chu H, Li G, Chan JF-W, To KK-W, Yang Z, Jiang S, Yuen K, Clevers H, Zhou J. 2023. Human airway and nasal organoids reveal escalating replicative fitness of SARS-CoV-2 emerging variants. Proc Natl Acad Sci USA 120:e2300376120. doi:10.1073/pnas.2300376120 37068258 PMC10151566

[29] Katsura H, Sontake V, Tata A, Kobayashi Y, Edwards CE, Heaton BE, Konkimalla A, Asakura T, Mikami Y, Fritch EJ, Lee PJ, Heaton NS, Boucher RC, Randell SH, Baric RS, Tata PR. 2020. Human lung stem cell-based alveolospheres provide insights into SARS-CoV-2-mediated interferon responses and pneumocyte dysfunction. Cell Stem Cell 27:890–904. doi:10.1016/j.stem.2020.10.005 33128895 PMC7577733

[30] Han Y, Duan X, Yang L, Nilsson-Payant BE, Wang P, Duan F, Tang X, Yaron TM, Zhang T, Uhl S, et al.. 2021. Identification of SARS-CoV-2 inhibitors using lung and colonic organoids. Nature 589:270–275. doi:10.1038/s41586-020-2901-9 33116299 PMC8034380

[31] Pei R, Feng J, Zhang Y, Sun H, Li L, Yang X, He J, Xiao S, Xiong J, Lin Y, Wen K, Zhou H, Chen J, Rong Z, Chen X. 2021. Host metabolism dysregulation and cell tropism identification in human airway and alveolar organoids upon SARS-CoV-2 infection. Protein Cell 12:717–733. doi:10.1007/s13238-020-00811-w 33314005 PMC7732737

[32] Wu X, Dao Thi VL, Huang Y, Billerbeck E, Saha D, Hoffmann HH, Wang Y, Silva LAV, Sarbanes S, Sun T, Andrus L, Yu Y, Quirk C, Li M, MacDonald MR, Schneider WM, An X, Rosenberg BR, Rice CM. 2018. Intrinsic immunity shapes viral resistance of stem cells. Cell 172:423–438. doi:10.1016/j.cell.2017.11.018 29249360 PMC5786493

[33] Gong Q, Jiang R, Ji L, Lin H, Liu M, Tang X, Yang Y, Han W, Chen J, Guo Z, Wang Q, Li Q, Wang X, Jiang T, Xie S, Yang X, Zhou P, Shi Z, Lin X. 2024. Establishment of a human organoid-based evaluation system for assessing interspecies infection risk of animal-borne coronaviruses. Emerg Microbes Infect 13:2327368. doi:10.1080/22221751.2024.2327368 38531008 PMC10967677

[34] Park D, Kim S, Jang H, Kim K, Ji HY, Yang H, Kwon W, Kang Y, Hwang S, Kim H, Casel MAB, Choi I, Yang J, Lee J, Choi YK. 2024. Differential beta‐coronavirus infection dynamics in human bronchial epithelial organoids. J Med Virol 96:e29600. doi:10.1002/jmv.29600 38591240

[35] Hysenaj L, Little S, Kulhanek K, Magnen M, Bahl K, Gbenedio OM, Prinz M, Rodriguez L, Andersen C, Rao AA, et al.. 2023. SARS-CoV-2 infection of airway organoids reveals conserved use of Tetraspanin-8 by Ancestral, Delta, and Omicron variants. Stem Cell Rep 18:636–653. doi:10.1016/j.stemcr.2023.01.011 PMC9948283 36827975

[36] Sharma K, Thacker VV, Dhar N, Clapés Cabrer M, Dubois A, Signorino-Gelo F, Mullenders J, Knott GW, Clevers H, McKinney JD. 2021. Early invasion of the bladder wall by solitary bacteria protects UPEC from antibiotics and neutrophil swarms in an organoid model. Cell Rep 36:109351. doi:10.1016/j.celrep.2021.109351 34289360

[37] Williamson IA, Arnold JW, Samsa LA, Gaynor L, DiSalvo M, Cocchiaro JL, Carroll I, Azcarate-Peril MA, Rawls JF, Allbritton NL, Magness ST. 2018. A high-throughput organoid microinjection platform to study gastrointestinal microbiota and luminal physiology. Cell Mol Gastroenterol Hepatol 6:301–319. doi:10.1016/j.jcmgh.2018.05.004 30123820 PMC6092482

[38] Li M, Ayyanathan K, Dittmar M, Miller J, Tapescu I, Lee JS, McGrath ME, Xue Y, Vashee S, Schultz DC, Frieman MB, Cherry S. 2023. SARS-CoV-2 ORF6 protein does not antagonize interferon signaling in respiratory epithelial Calu-3 cells during infection. mBio 14:e0119423. doi:10.1128/mbio.01194-23 37377442 PMC10470815

[39] Shapira T, Monreal IA, Dion SP, Buchholz DW, Imbiakha B, Olmstead AD, Jager M, Désilets A, Gao G, Martins M, et al.. 2022. A TMPRSS2 inhibitor acts as a pan-SARS-CoV-2 prophylactic and therapeutic. Nature 605:340–348. doi:10.1038/s41586-022-04661-w 35344983 PMC9095466

[40] Chen X, Saccon E, Appelberg KS, Mikaeloff F, Rodriguez JE, Vinhas BS, Frisan T, Végvári Á, Mirazimi A, Neogi U, Gupta S. 2021. Type-I interferon signatures in SARS-CoV-2 infected Huh7 cells. Cell Death Discov 7:114. doi:10.1038/s41420-021-00487-z 34006825 PMC8129603

[41] Millet JK, Whittaker GR. 2014. Host cell entry of Middle East respiratory syndrome coronavirus after two-step, furin-mediated activation of the spike protein. Proc Natl Acad Sci USA 111:15214–15219. doi:10.1073/pnas.1407087111 25288733 PMC4210292

[42] Shirato K, Kawase M, Matsuyama S. 2013. Middle East respiratory syndrome coronavirus infection mediated by the transmembrane serine protease TMPRSS2. J Virol 87:12552–12561. doi:10.1128/JVI.01890-13 24027332 PMC3838146

[43] Jin Z, Shao Z, Yang S, Guo A, Han Y, Wu Y, Zhao Y, Wu Y, Shen J, Zhang M, Zhan X, Diao W, Ying S, Zhang C, Li W, Shen H, Chen Z, Yan F. 2023. Airway epithelial cGAS inhibits LPS-induced acute lung injury through CREB signaling. Cell Death Dis 14:844. doi:10.1038/s41419-023-06364-0 38114479 PMC10730695

[44] Nova Z, Skovierova H, Strnadel J, Halasova E, Calkovska A. 2020. Short-term versus long-term culture of a549 cells for evaluating the effects of lipopolysaccharide on oxidative stress, surfactant proteins and cathelicidin LL-37. Int J Mol Sci 21:1148. doi:10.3390/ijms21031148 32050475 PMC7036965

[45] De S, Zhou H, DeSantis D, Croniger CM, Li X, Stark GR. 2015. Erlotinib protects against LPS-induced endotoxicity because TLR4 needs EGFR to signal. Proc Natl Acad Sci USA 112:9680–9685. doi:10.1073/pnas.1511794112 26195767 PMC4534288

[46] Giobbe GG, Bonfante F, Jones BC, Gagliano O, Luni C, Zambaiti E, Perin S, Laterza C, Busslinger G, Stuart H, Pagliari M, Bortolami A, Mazzetto E, Manfredi A, Colantuono C, Di Filippo L, Pellegata AF, Panzarin V, Thapar N, Li VSW, Eaton S, Cacchiarelli D, Clevers H, Elvassore N, De Coppi P. 2021. SARS-CoV-2 infection and replication in human gastric organoids. Nat Commun 12:6610. doi:10.1038/s41467-021-26762-2 34785679 PMC8595698

[47] Ekanger CT, Zhou F, Bohan D, Lotsberg ML, Ramnefjell M, Hoareau L, Røsland GV, Lu N, Aanerud M, Gärtner F, Salminen PR, Bentsen M, Halvorsen T, Ræder H, Akslen LA, Langeland N, Cox R, Maury W, Stuhr LEB, Lorens JB, Engelsen AST. 2022. Human organotypic airway and lung organoid cells of bronchiolar and alveolar differentiation are permissive to infection by influenza and SARS-CoV-2 respiratory virus. Front Cell Infect Microbiol 12:841447. doi:10.3389/fcimb.2022.841447 35360113 PMC8964279

[48] Stölting H, Baillon L, Frise R, Bonner K, Hewitt RJ, Molyneaux PL, Gore ML, Barclay WS, Saglani S, Lloyd CM, Breathing Together Consortium. 2022. Distinct airway epithelial immune responses after infection with SARS-CoV-2 compared to H1N1. Mucosal Immunol 15:952–963. doi:10.1038/s41385-022-00545-4 35840680 PMC9284972

[49] Chen J, Yang X, Si H, Gong Q, Que T, Li J, Li Y, Wu C, Zhang W, Chen Y, et al.. 2023. A bat MERS-like coronavirus circulates in pangolins and utilizes human DPP4 and host proteases for cell entry. Cell 186:850–863. doi:10.1016/j.cell.2023.01.019 36803605 PMC9933427

[50] Chu DKW, Hui KPY, Perera RAPM, Miguel E, Niemeyer D, Zhao J, Channappanavar R, Dudas G, Oladipo JO, Traoré A, et al.. 2018. MERS coronaviruses from camels in Africa exhibit region-dependent genetic diversity. Proc Natl Acad Sci USA 115:3144–3149. doi:10.1073/pnas.1718769115 29507189 PMC5866576

[51] Alsamman AM, Zayed H. 2020. The transcriptomic profiling of SARS-CoV-2 compared to SARS, MERS, EBOV, and H1N1. PLoS One 15:e0243270. doi:10.1371/journal.pone.0243270 33301474 PMC7728291

[52] Schroeder S, Mache C, Kleine-Weber H, Corman VM, Muth D, Richter A, Fatykhova D, Memish ZA, Stanifer ML, Boulant S, Gultom M, Dijkman R, Eggeling S, Hocke A, Hippenstiel S, Thiel V, Pöhlmann S, Wolff T, Müller MA, Drosten C. 2021. Functional comparison of MERS-coronavirus lineages reveals increased replicative fitness of the recombinant lineage 5. Nat Commun 12:5324. doi:10.1038/s41467-021-25519-1 34493730 PMC8423819

[53] Kim ES, Choe PG, Park WB, Oh HS, Kim EJ, Nam EY, Na SH, Kim M, Song KH, Bang JH, Park SW, Kim HB, Kim NJ, Oh MD. 2016. Clinical progression and cytokine profiles of middle east respiratory syndrome coronavirus infection. J Korean Med Sci 31:1717–1725. doi:10.3346/jkms.2016.31.11.1717 27709848 PMC5056202

[54] de Wilde AH, Raj VS, Oudshoorn D, Bestebroer TM, van Nieuwkoop S, Limpens RWAL, Posthuma CC, van der Meer Y, Bárcena M, Haagmans BL, Snijder EJ, van den Hoogen BG. 2013. MERS-coronavirus replication induces severe in vitro cytopathology and is strongly inhibited by cyclosporin A or interferon-α treatment. J Gen Virol 94:1749–1760. doi:10.1099/vir.0.052910-0 23620378 PMC3749523

[55] Li K, Wohlford-Lenane C, Bartlett JA, McCray PB. 2022. Inter-individual variation in receptor expression influences MERS-CoV infection and immune responses in airway epithelia. Front Public Health 9:756049. doi:10.3389/fpubh.2021.756049 35059374 PMC8763803

[56] Hocke AC, Becher A, Knepper J, Peter A, Holland G, Tönnies M, Bauer TT, Schneider P, Neudecker J, Muth D, Wendtner CM, Rückert JC, Drosten C, Gruber AD, Laue M, Suttorp N, Hippenstiel S, Wolff T. 2013. Emerging human middle East respiratory syndrome coronavirus causes widespread infection and alveolar damage in human lungs. Am J Respir Crit Care Med 188:882–886. doi:10.1164/rccm.201305-0954LE 24083868

[57] Alsaad KO, Hajeer AH, Al Balwi M, Al Moaiqel M, Al Oudah N, Al Ajlan A, AlJohani S, Alsolamy S, Gmati GE, Balkhy H, Al-Jahdali HH, Baharoon SA, Arabi YM. 2018. Histopathology of Middle East respiratory syndrome coronovirus (MERS-CoV) infection - clinicopathological and ultrastructural study. Histopathology 72:516–524. doi:10.1111/his.13379 28858401 PMC7165512

[58] Banerjee A, Nasir JA, Budylowski P, Yip L, Aftanas P, Christie N, Ghalami A, Baid K, Raphenya AR, Hirota JA, Miller MS, McGeer AJ, Ostrowski M, Kozak RA, McArthur AG, Mossman K, Mubareka S. 2020. Isolation, sequence, infectivity, and replication kinetics of severe acute respiratory syndrome coronavirus 2. Emerg Infect Dis 26:2054–2063. doi:10.3201/eid2609.201495 32558639 PMC7454076

